# Cardiac resynchronization therapy-heart failure (CRT-HF) clinic: A novel model of care

**DOI:** 10.1371/journal.pone.0222610

**Published:** 2019-09-19

**Authors:** Eiran Z. Gorodeski, Christina Magnelli-Reyes, Laurie Ann Moennich, Adam Grimaldi, John Rickard

**Affiliations:** 1 Section of Heart Failure and Cardiac Transplantation, Department of Cardiovascular Medicine, Heart and Vascular Institute, Cleveland Clinic, Cleveland, Ohio, United States of America; 2 Section of Cardiac Pacing and Electrophysiology, Department of Cardiovascular Medicine, Heart and Vascular Institute, Cleveland Clinic, Cleveland, Ohio, United States of America; 3 Heart and Vascular Research, Heart and Vascular Institute, Cleveland Clinic, Cleveland, Ohio, United States of America; University Medical Center Groningen, University of Groningen, NETHERLANDS

## Abstract

**Background:**

Post-implant care of patients with heart failure (HF) undergoing cardiac resynchronization therapy (CRT) is not addressed in current HF or CRT guidelines and is often fragmented with poor communication between specialties. We sought to develop a new model of post-CRT care which could be implemented in busy clinical settings.

**Methods and results:**

We designed a novel, multidisciplinary approach to standardizing post CRT care. All patients receiving a CRT device at the Cleveland Clinic between March 2017 and August 2018 were invited to be seen in the clinic 6 months post implant. A one-time collaborative visit encompassing cardiac imaging, heart failure, and electrophysiology care was performed. We recorded the operational feasibility of the clinic in terms of patient throughput as well as patient characteristics, interventions, and new diagnoses made. Between September 2017 and February 2019, 150 patients were seen in the clinic. Of these, 125 patients had their index CRT implanted for standard indications and were included in the current analysis. Approximately 45 minutes were dedicated for each patient visit. Interventions in care were made in 95% of patients, with CRT non-responders offered a higher number of interventions as compared to responders (median 3 versus 2 interventions). Types of interventions were device-related (26% of population), medication-related (74%), and referral for alternate medical services (80%).

**Conclusions:**

Multidisciplinary post-implant care of patients with HF receiving CRT devices, regardless of CRT response status, is feasible and results in frequent medical interventions.

## Introduction

Cardiac resynchronization therapy (CRT) is a guideline-recommended therapy for select patients with heart failure with reduced ejection fraction (HFrEF). In multiple large-scale clinical trials CRT was shown to improve quality of life and functionality, induce reverse left ventricular remodeling, reduce heart failure hospitalizations, and improve mortality in patients with HFrEF and QRS widening on a 12-lead ECG [1–4].

Medical management of patients receiving CRT is complex. In addition to left ventricular dysfunction of a variety of etiologies, appropriate CRT candidates often also have multiple comorbidities and frailty [5]. Additionally, outcomes following CRT device implantation are heterogeneous ranging from marked improvements in clinical status and left ventricular function, to rapid deterioration. As such, longitudinal care of patients with CRT commonly requires coordinated expertise in electrophysiology, heart failure (HF), and cardiac imaging.

Multiple medical societies have published guidelines outlining appropriate use of CRT [6–8]. All guidelines, however, stop at CRT implantation and fail to provide guidance for appropriate post-implantation management. As such, post-CRT care tends to be fragmented and suboptimal. To address this deficiency, we collaboratively created the Cardiac Resynchronization Therapy-Heart Failure (CRT-HF) clinic at our institution, a novel model of care which standardizes post-CRT follow-up and coordinates a multidisciplinary approach with the goal of optimizing outcomes in this challenging patient population. We sought to develop a model that could be achieved in a wide variety of practice care settings. In the current paper we describe the design, implementation, and our initial observations of this model, with a specific focus on interventions provided at the time of CRT-HF clinic visit.

## Methods

### CRT-HF clinic overview

This study was approved by the Institutional Review Board at Cleveland Clinic. Written informed consent was obtained from all study participants. The CRT-HF clinic is a multi-disciplinary clinic jointly run by a cardiac electrophysiologist and a HF cardiologist, which aims to see all patients receiving a CRT device (LV lead or His bundle lead) for a one-time visit approximately 6 months after implantation. The mission of the clinic is to identify patients who are at high risk for poor outcomes earlier in their disease course, and to intensify their management in hopes of improving outcomes. The goals of the clinic are to deliver optimally timed, standardized interventions to all patients with symptomatic HF who received CRT pacemakers or pacemakers/defibrillators, by optimizing HF guideline-directed pharmacological therapy, troubleshooting commonly recognized device issues, addressing comorbidities, and referring for invasive interventions when appropriate. Specific goals include:

- To standardize approaches to CRT device management, including interrogation and optimization.
- To more rapidly identify patients who fail to improve, and onboard them to appropriate longitudinal care, invasive hemodynamic assessment and/or early evaluation for advanced HF options (pulmonary artery pressure monitoring, cardiac transplantation, or left ventricular assist device).
- To maximize clinical benefits in patients who have realized clinical improvement with CRT pacing.

### Recruitment of patients

In an effort to improve appointment adherence patients are informed about the CRT-HF clinic at several points in time. First, all patients scheduled to have a CRT device implanted are informed about the CRT-HF clinic by their implanting electrophysiologist before implantation occurs. Second, within 24 hours of device implantation and before hospital discharge patients are again informed about the clinic by device clinic staff, and reading material is given. Finally, several months later and closer to the date of the visit patients receive a phone call from a scheduling team which finalizes the visit date and confirms attendance.

### Clinic workflow

**Fig 1:**
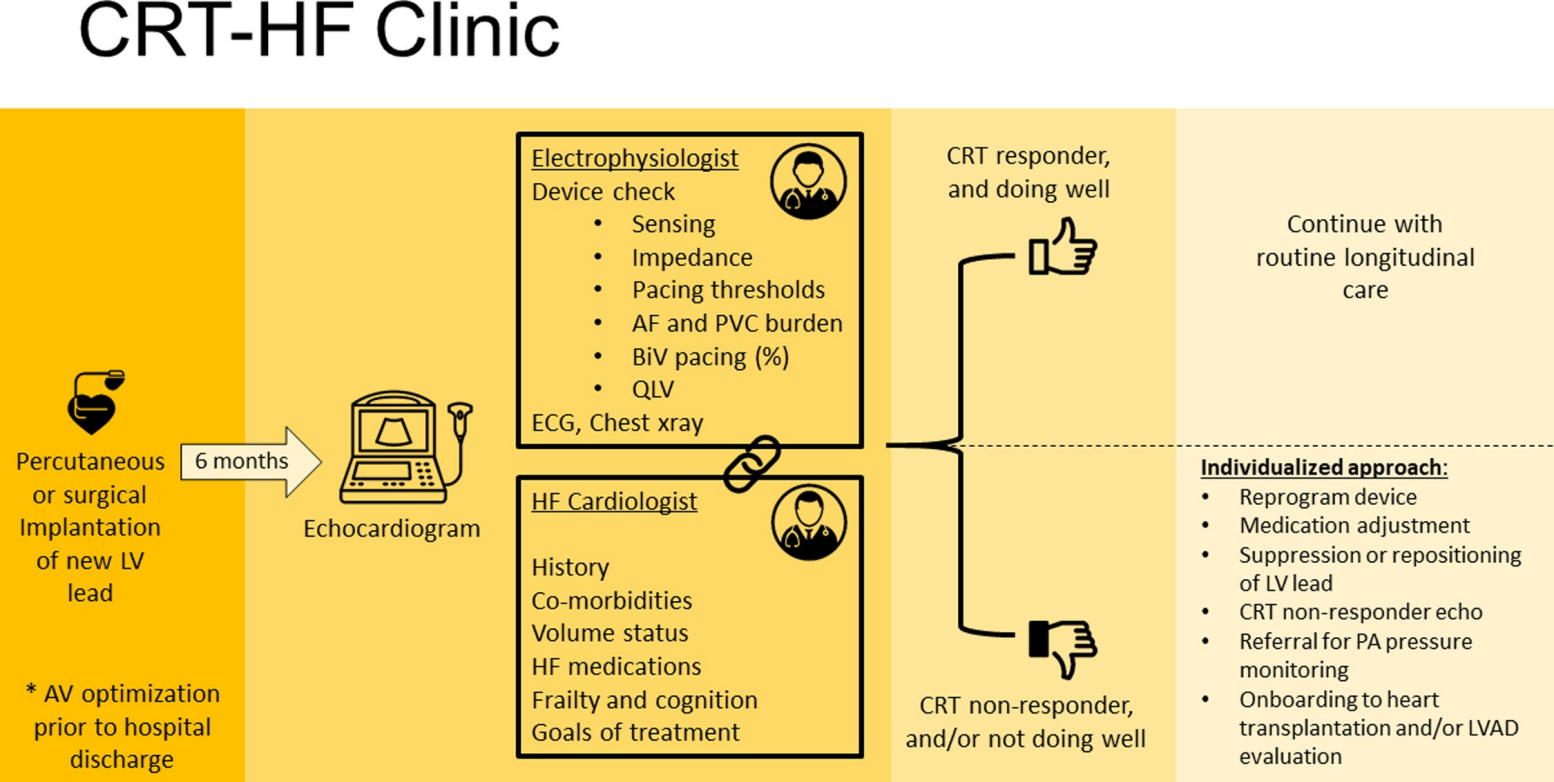
CRT-HF clinic workflow.

An overview of the CRT-HF clinic is shown in **Fig 1**. On the day of the CRT-HF clinic visit, patients first undergo an echocardiogram to assess LV response to CRT pacing. Echocardiographers interpret the results, with special attention paid to evaluation of left ventricular volumes, left ventricular ejection fraction (LVEF), and diastolic filling patterns. Based on changes between the pre- and post-CRT echocardiograms patients are classified as “responders” and “non-responders”. A “responder” is defined as a patient with absolute LVEF improvement ≥5% coupled with a reduction in left ventricular end-systolic volume ≥10%. A “non-responder” is classified as any patient not meeting this definition. Patients are classified as above given the association of these classifications with long term outcomes based on previously published data [9].

Patients then check into clinic, and a nurse checks vital signs, reconciles medications, and performs a battery of tests including assessment of quality of life (EQ-5D), functionality (six minute walk test), cognition (the Mini-Cog), and frailty (gait speed, get up and go test, and handgrip strength using a Jamar hydraulic hand dynamometer) [10–13].

Immediately prior to seeing the patient, a multi-disciplinary team including an electrophysiologist, a HF cardiologist, and the clinic nurse review each patient’s case together. Data reviewed include the patient’s past medical and cardiac history, current medication regimen, the original goal of CRT (e.g., improvement of LV function versus preservation of LV function in the setting of a need for ventricular pacing due to bradycardia), pre-implantation ECG for underlying electrical substrate (QRS morphology and duration prior to CRT), post-implantation ECG, CRT lead location on a posterior-anterior and lateral chest X-ray, coronary sinus venography from the implantation, and any complicating issues from the case. The team also reviews prior and current echocardiograms, and the quality of life, functionality, cognition, and frailty data collected by the nurse.

Each patient is then seen by both physicians simultaneously in an exam room that has an interview area with computers, an exam table, ECG machine, and device interrogators. The HF cardiologist conducts a medical interview while the electrophysiologist simultaneously performs a full device check. The medical interview is comprised of questions about the patient’s current status and subjective functionality, perceived response to CRT pacing, prior or recent HF hospitalizations, and compliance with and tolerance of cardiac medications. The device check entails sensing and pacing thresholds, and impedances for all leads (no sensing for the CRT lead). QLV is recorded by suppressing pacing and measuring the time from the onset of the far-field electrogram (typically can to RV coil) to the first sharp deflection on the local electrogram on the LV lead. In patients with no underlying ventricular rhythm QLV is measured compared to the right ventricular paced rhythm. Percent biventricular pacing, atrial fibrillation burden, and PVC burden are also recorded. Anodal stimulation is tested for when clinically suspected. If the patient is not device-dependent, we perform an ECG while suppressing pacing to assess underlying electrical substrate, if a suppressed ECG is not already available. A complete physical exam is then performed by both physicians.

The team then exits the room to discuss the case and develop an individualized patient plan. The team then re-enters the room and both physicians discuss the suggested plan with the patient. When obvious device-based issues are present, they are corrected when possible in clinic. The plan of care is communicated to each patient’s primary clinicians and implemented in a collaborative way. The CRT-HF clinic appointment flow described here is offered only once for each patient, and all subsequent longitudinal follow-up is done with each patient’s primary clinicians as indicated.

### Research registry and data analysis

Data regarding all patients seen in the CRT-HF clinic was collected in a prospective manner and maintained in a secure REDCap database. Entry into the registry required signed informed consent. Creation and maintenance of this registry, as well as analyses related to it, were approved by the Institutional Review Board at Cleveland Clinic.

For the purposes of this study demographic and clinical variables were stratified by CRT response status, as defined above. Continuous variables are presented as mean and standard deviation, and categorical variables as frequencies and percentages. The number of interventions provided in the CRT-HF clinic are presented in a density graph, with point summaries as median. Analyses were performed using R version 3.5.3 (www.r-project.org). We used Wickham’s ggplot2 library version 3.1.0 (www.ggplot2.org) for graphics.

## Results

### Patient characteristics

**Fig 2:**
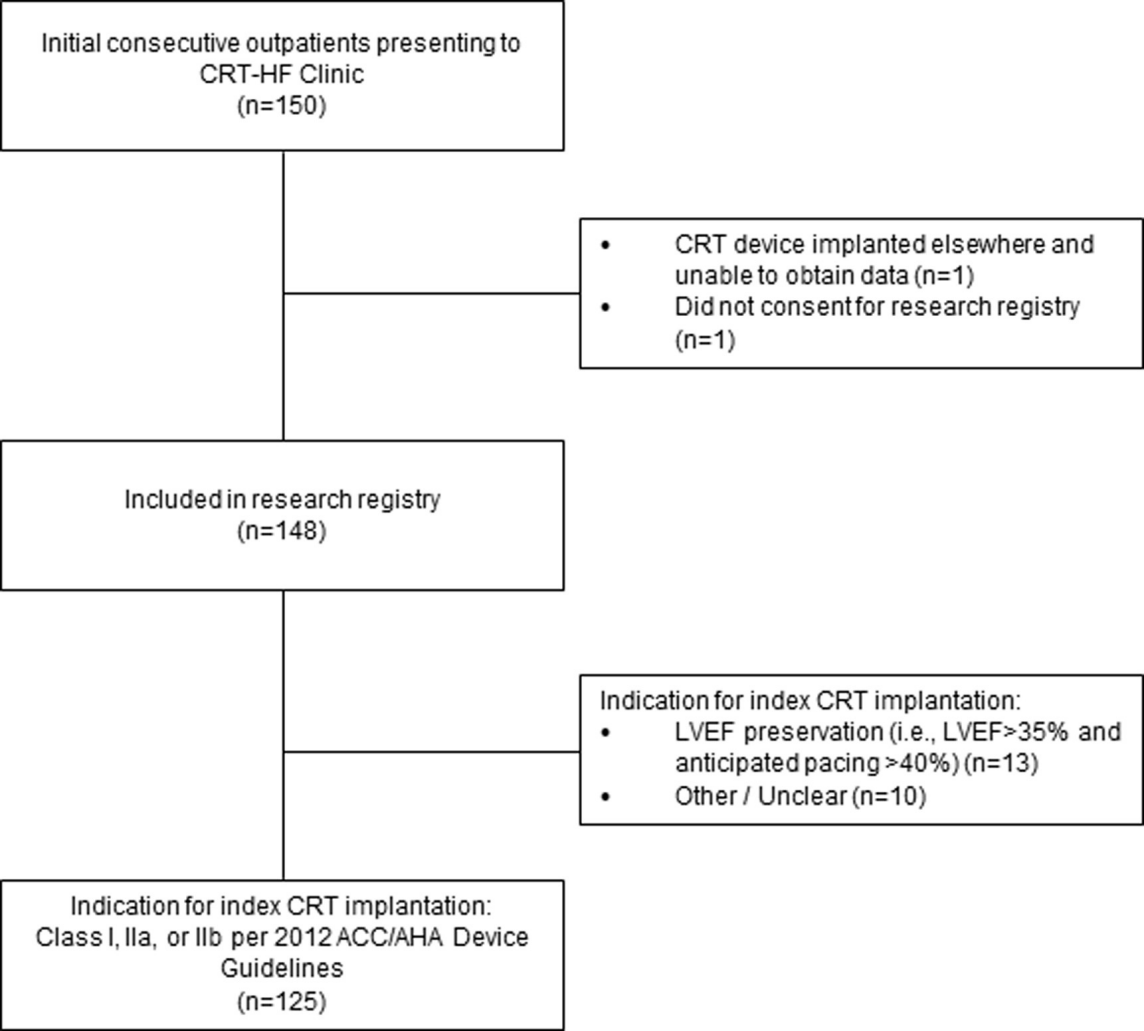
CONSORT diagram.

We implemented the CRT-HF Follow-up Clinic at Cleveland Clinic Main Campus, Cleveland, OH, in September 2017. Between September 2017 and February 2019 we held a median of 2 clinic sessions per month (range 1–3), and were able to see a total of 150 patients. The characteristics of patients who met criteria for inclusion in our research registry as well as standard indication for CRT implantation (n = 125) are shown in the CONSORT diagram (**Fig 2**), and their characteristics are shown in **Table 1**. On average, the amount of time between CRT implantation and presentation at the CRT-HF Clinic was 6.7±1.4 months. Clinic visits lasted approximately 45 minutes each. Patients tended to be older white men, with a variety of cardiovascular comorbidities. A large majority had non-ischemic cardiomyopathy, with symptomatic Stage C HFrEF. There were no clinically meaningful differences in patient characteristics between CRT responders and non-responders (**Table 1**).

**Table 1:** Characteristics of outpatients presenting to CRT-HF clinic.

	Responders (n = 76)	Non-Responders (n = 49)	p
**Age, years, mean (SD)**	69 (13)	67 (13)	0.36
**Male, n (%)**	40 (53)	35 (71)	0.06
**Body mass index, kg/m**^**2**^**, mean (SD)**	29 (7)	31 (9)	0.31
**Race, n (%)**			0.18
Black	9 (12)	7 (14)	
Other	0 (0)	2 (4)	
White	67 (88)	40 (82)	
**Non-ischemic cardiomyopathy, n (%)**	56 (74)	30 (61)	0.20
**ACC/AHA heart failure stage, n (%)**			1.00
B	4 (5)	5 (10)	
C	72 (95)	42 (86)	
D	0 (0)	2 (4)	
**New York Heart Association class at time of CRT-HF clinic visit, n (%)**			1.00
I	15 (20)	7 (14)	
II	38 (50)	25 (51)	
III	23 (30)	11 (22)	
IV	0 (0)	6 (12)	
**Medical history, n (%)**			
Hypertension	56 (74)	34 (69)	0.75
Diabetes mellitus	31 (41)	11 (22)	0.05
Myocardial infarction	18 (24)	16 (33)	0.37
Atrial fibrillation or flutter	30 (39)	25 (51)	0.28
Ventricular tachycardia	14 (18)	15 (31)	0.17
Peripheral arterial disease	1 (1)	3 (6)	0.33
Transient ischemic attack	5 (7)	1 (2)	0.47
Stroke	6 (8)	5 (10)	0.90
Chronic obstructive pulmonary disease or Emphysema	5 (7)	8 (16)	0.15
Depression	15 (20)	3 (6)	0.06
Dementia	3 (4)	0 (0)	0.42
Hypothyroidism	12 (16)	8 (16)	1.00
Chronic kidney disease			0.23
None	63 (83)	38 (78)	
Mild renal insufficiency (GFR 60–89)	2 (3)	0 (0)	
Moderate renal insufficiency (GFR 30–59)	7 (9)	9 (18)	
Severe renal insufficiency (GFR 15–29)	4 (5)	1 (2)	
Renal failure (GFR < 15 ml/min or patient on HD)	0 (0)	1 (2)	
**Heart failure medications**			
Beta blocker	74 (97)	44 (90)	0.16
ACE inhibitor	28 (37)	18 (37)	1.00
Angiotensin receptor blocker	17 (22)	8 (16)	0.55
Loop diuretic	57 (75)	33 (67)	0.47
Thiazide diuretic	3 (4)	3 (6)	0.90
Mineralocorticoid receptor antagonist	29 (38)	27 (55)	0.09
Vasodilator	18 (24)	11 (22)	1.00
Angiotensin Receptor-Neprilysin Inhibitor	16 (21)	11 (22)	1.00
**Functionality and Quality of Life**			
Six minute walk distance, feet, mean (SD)	1076 (482)	1066 (377)	0.90
Problems with mobility9 (EQ-5D), n (%)	36 (47)	24 (49)	1.00
Problems with self-care (EQ-5D), n (%)	10 (13)	6 (12)	1.00
Problems with usual activities (EQ-5D), n (%)	22 (29)	16 (33)	0.81
Problems with pain or discomfort (EQ-5D), n (%)	34 (45)	22 (45)	1.00
Problems with anxiety or depression (EQ-5D), n (%)	25 (33)	12 (24)	0.42
Visual analogue scale*, mean (SD)	72 (17)	71 (18)	0.63

### CRT characteristics and outcomes

Indications for index CRT implantation, as adjudicated retrospectively during CRT-HF clinic visit, are shown in **Table 2**. As compared to non-responders, CRT responders were more likely to have had a Class I indication for their device implantation. Further, presence of LBBB on ECG prior to CRT implantation was more likely to have been present in CRT responders, while presence of non-specific IVCD was more likely to have been present in CRT non-responders. The echocardiographic variables before and after CRT implantation, stratified by response status, are shown in **Table 3**.

**Table 2:** CRT implant characteristics.

	Responders (n = 76)	Non-Responders (n = 49)	p
Indications for CRT Therapy^*^, n (%)			0.01
Class I	39 (51)	16 (33)	
Class IIa	35 (46)	25 (51)	
Class IIb	2 (3)	8 (16)	
**QRS morphology prior to CRT implantation, n (%)**			
Left bundle branch block	53 (70)	22 (45)	0.01
Right bundle branch block	0 (0)	3 (6)	0.11
Non-specific interventricular conduction delay	4 (5)	14 (29)	<0.01
Paced	5 (7)	1 (2)	0.47
Narrow	0 (0)	2 (4)	0.30
Complete heart block	14 (18)	7 (14)	0.72
**CRT type, n (%)**			1.00
CRT-D	54 (71)	34 (69)	
CRT-P	22 (29)	15 (31)	
**Lead type, n (%)**			1.00
LV lead	72 (95)	47 (96)	
HIS lead	4 (5)	2 (4)	
**LV lead position, PA chest xray**^**^**^**, n (%)**			0.37
Apical	11 (15)	10 (21)	
Mid	49 (68)	33 (70)	
Basal	12 (17)	4 (9)	
**LV lead position, lateral chest xray**^**^**^**, n (%)**			0.77
Anterolateral	1 (1)	0 (0)	
Lateral	16 (22)	12 (26)	
Posterolateral	52 (72)	34 (72)	
Posterior	3 (4)	1 (2)	

* Indications for CRT therapy per ACC/AHA 2012 Device Guidelines

^^^ Excludes 6 patients who had HIS leads placed

**Table 3:** Echocardiographic variables before CRT implantation and at time of CRT-HF clinic visit.

	Responders (n = 76)				Non-responders (n = 49)			
	Pre-CRT	Missing	Post-CRT	Missing	Pre-CRT	Missing	Post-CRT	Missing
Left ventricular ejection fraction, %, mean (SD)	28 (7)	0%	43 (9)	0%	26 (6)	0%	27 (7)	0%
Left ventricular end-diastolic volume, mL, mean (SD)	180 (54)	13%	137 (43)	4%	217 (68)	14%	218 (73)	6%
Left ventricular end-systolic volume, mL, mean (SD)	135 (48)	20%	85 (37)	8%	158 (55)	20%	164 (60)	10%

### Patient management at the CRT-HF clinic

**Fig 3:**
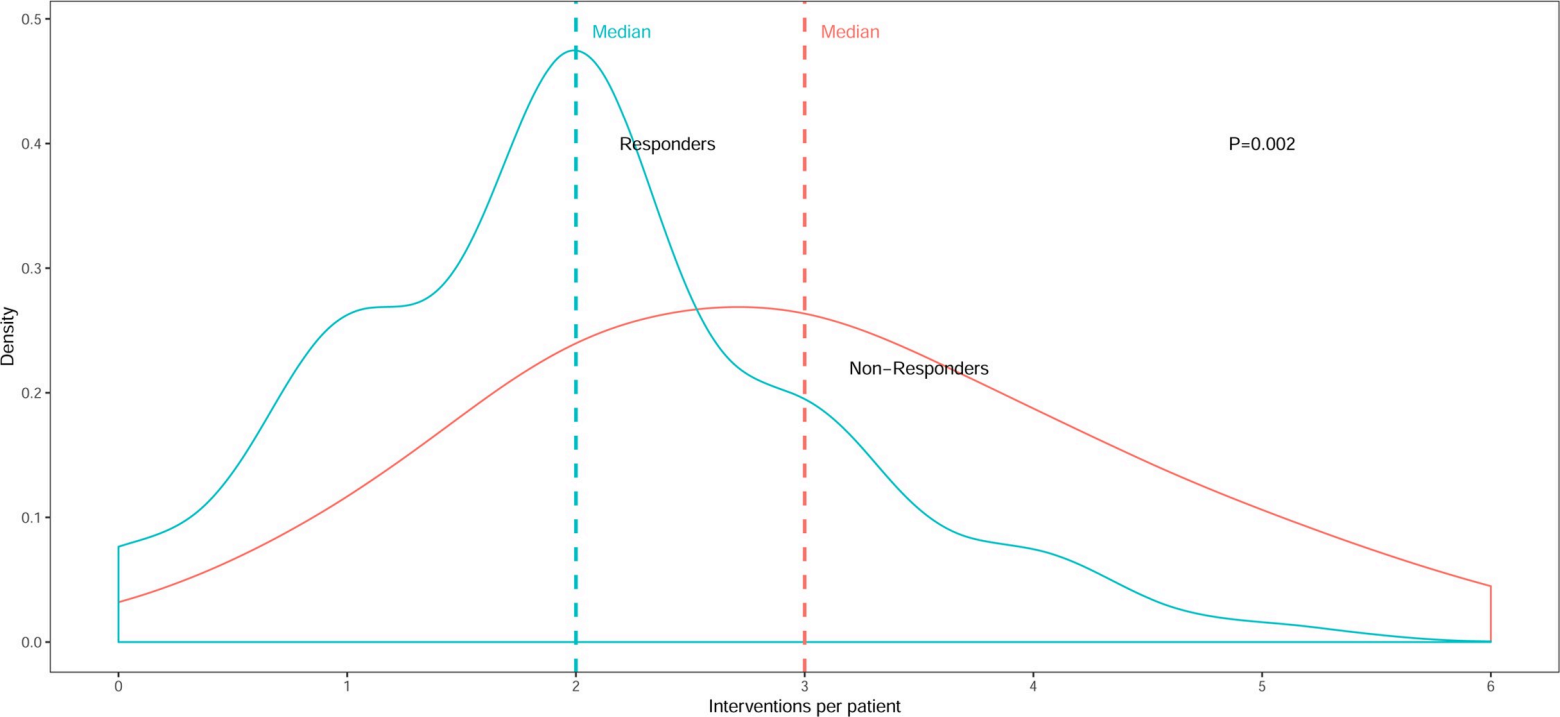
Medical interventions provided at the CRT-HF Clinic, stratified by CRT response status.

A large majority of patients (95%) seen in the CRT-HF clinic were offered one or more medical intervention at time of clinic visit. The median number of interventions per patient was 2 (range 0–6) for the entire cohort, with non-responders offered a significantly higher number of interventions than responders (median 3 versus 2 interventions) (**Fig 3**).

Types of interventions provided at the CRT-HF clinic are shown in **Table 4**, with 26% of patients having device-related changes, 74% having medication-related changes, and 80% having other types of interventions. CRT non-responders were more likely to be referred for advanced multi-modality imaging and have their pacing vector changed, as compared to CRT responders. Regardless of CRT response status, all patients were likely to have their medication regimen optimized, most frequently having heart failure medication doses increased or new ones added. CRT non-responders were more likely to be offered referral for more intensive longitudinal cardiac care, including advanced HF staging, with 4 patients being referred for implantable pulmonary artery pressure monitoring.

**Table 4:** Medical interventions provided at the CRT-HF clinic.

	Responders (n = 76)	Non-Responders (n = 49)	p
**Device/pacing-related interventions, n (%)**			
Withheld biventricular pacing	1 (1.3)	4 (8.2)	0.15
Referred for advanced multi-modality imaging	2 (2.6)	6 (12.2)	0.08
Referred for lead reposition	2 (2.6)	3 (6.1)	0.61
Referred for placement of LV lead	0 (0.0)	1 (2.0)	0.82
Changed pacing vector	5 (6.6)	11 (22.4)	0.02
Made other changes in device programming	2 (2.6)	1 (2.0)	1.00
*None*	*64 (84*.*2)*	*29 (59*.*2)*	*<0*.*01*
**Medication-related interventions, n (%)**			
Decrease dose of current medications	9 (11.8)	4 (8.2)	0.72
Increase dose of current medication	22 (28.9)	13 (26.5)	0.93
Switch to new HF medication	8 (10.5)	11 (22.4)	0.12
Prescribed new cardiac medication	10 (13.2)	13 (26.5)	0.10
Pharmacologic recommendation made to another provider	19 (25.0)	15 (30.6)	0.63
Stopped cardiac medication	6 (7.9)	5 (10.2)	0.90
*None*	*23 (30*.*3)*	*10 (20*.*4)*	*0*.*31*
**Other interventions, n (%)**			
Referred for advanced heart failure staging	3 (3.9)	8 (16.3)	0.04
Referred for implantable pulmonary artery pressure monitor	0 (0.0)	4 (8.2)	0.04
Referred to other medical services	21 (27.6)	10 (20.4)	0.48
Recommend follow up in HF clinic, or for care with HF pharmacist or HF advanced practice provider	53 (69.7)	35 (71.4)	1.00
Made new cardiac diagnoses with actionable interventions	8 (10.5)	12 (24.5)	0.07

## Discussion

We describe the design, operations, and impact of a novel multidisciplinary model of care for patients with HFrEF and CRT devices. This model addresses an aspect of care which is currently not represented in published device and HF guidelines: longitudinal management of this complex patient population. Our primary findings are that (1) operationalizing an all-comers CRT-HF clinic in a busy real-world clinical practice is feasible, (2) delivering simultaneous multidisciplinary care from an electrophysiologist and HF cardiologist in this ambulatory outpatient setting is feasible and is well received by patients, and (3) there are a variety of device-related and/or medical opportunities to improve care in essentially all patients who are 6-months out from CRT implantation, regardless of CRT response status.

The care of patients receiving CRT devices presents a unique challenge. By definition, such patients are arguably amongst the sickest patients most cardiologists care for. In addition, care for such patients requires a collaborative effort between heart failure cardiologists, electrophysiologists, and often imaging specialists. Oftentimes, heart failure cardiologists are not well versed in device programming and diagnostics. Similarly, electrophysiologists are often not ideal at managing advanced heart failure. Given these issue, suboptimal care is likely to result unless excellent communication between specialties is achieved.

In 2009, Mullens et al. described the first experience with a dedicated CRT clinic [14]. The authors found that patients in whom interventions could be made achieved superior outcomes compared to those where no interventions could be performed. This clinic was referral-based and therefore saw only a fraction of the total patients receiving CRT. While this model is attractive in that it is not overly resource intensive, it has several major problems. First, because it is referral-dependent many patients who could be helped in the clinic are never seen. Secondly, patients are often referred years after their CRT device was placed frequently missing a window where intervention could have been more effective.

In 2012, Altman et al. described a multidisciplinary care pathway approach for patients with CRT and HF [15]. The authors employed a “care pathway” approach—different than the Mullens “non-responder clinic” approach [14]—in which all patients at the institution receiving CRT were systematically followed. In this model patients were seen at 1-, 3-, and 6-months post-CRT implant, with at least two echocardiographic-guided optimizations. This strategy was shown to improve survival free of HF hospitalization and death compared to traditional non-multidisciplinary follow up. Despite these results, this model failed to gain traction in other centers likely because of the high number of echocardiographic tests and visits required.

We believe that the CRT-HF clinic model described here can be successfully implemented in a wide variety of academic and community settings. First, visits take approximately 45 minutes per patient, a time requirement that is not onerous. Second, two cardiologists are able to see the patient simultaneously, a practice that is efficient and encourages excellent communication and tight collaboration. Third, from an economic standpoint, both physicians are able to bill insurance separately for their services, a practice which addresses current hospital reimbursement requirements. In addition, a device check, pre-visit echocardiogram, and 6 minute hall test are also reimbursable. Despite two co-pays, the patient satisfaction with the clinic has been excellent as patients have appreciated the back and forth communication between the specialists face to face. In future value-based care settings the CRT-HF model of care would likely be even more advantageous.

Disparities in care for patients with chronic HF, especially as they relate to lack of adherence to guideline-directed therapies, have been well documented in the literature [16]. Our experience seeing patients with HFrEF who were 6-months out post-CRT implant demonstrates that tremendous opportunities exist to optimize the HF medication regimen in this population (**Table 4**). We observed many patients who were either not prescribed guideline-directed HF medications, were on suboptimal doses of appropriate HF medications, or had not been switched to the newest medical therapies including sacubitril-valsartan. Further, we were able to troubleshoot common device-related problems and determine common reasons for sub-optimal response. In some instances, invasive repositioning of the LV lead was recommended. In others, the LV lead was turned off entirely.

Going forward there are a number of novel factors that this care model would be well equipped to explore. One such area is that of chronotropic incompetence in this population. While randomized controlled trial data is largely lacking, cohort studies have suggested a role for cardiopulmonary stress testing in CRT recipients in identifying chronotropic incompetence (CI) as well as heart rates exceeding the upper rate during moderate exercise.[17] Whether targeting CI or excessive heart rates during moderate exercise can improve outcomes in CRT recipients is unclear. Secondly, it is being increasingly recognized that the terms “responder” and “non-responder” may be inappropriate as they fail to take into account the natural history of disease in any individual patient. It has increasingly been recognized that there is a subset of patients who may not necessarily improve in terms of LVESV reduction but stabilize what was a steady downhill course in terms of adverse remodeling. The term “non-progressor” has been used to describe patients such as this. The use of standardized scores pre-implant may be helpful in predicting what the expected LVESV reduction with CRT would have been and thus help define phenotypes such as the “non-progressor”.[18] Standardization of follow up in a multidisciplinary fashion may help answer these and other questions. Lastly, an often overlooked goal would be to make a “responder” into a “super-responder”. While this certainly is not feasible in everyone, many responders have opportunities to improve further. Structured exercise training, for example, has been shown to further improve exercise capacity, hemodynamic, measures, and quality of life in patients who have done well with CRT.[19]

Our study has several limitations. First, it is not yet known whether the CRT-HF clinic model described here can improve downstream patient outcomes. We intend on tracking outcomes of our patient cohort and will be able to report this in the future. Second, we implemented the CRT-HF clinic in our hospital, which is a tertiary referral center. Whether our findings will be generalizable to patients in other settings is unknown. Third, as our study is observational in nature and lacks a control group it is unknown if changes in management would have been made otherwise.

## Supporting information

S1 File Table data. (CSV)
